# Identifying the role of transient receptor potential channels (TRPs) in kidney renal clear cell carcinoma and their potential therapeutic significances using genomic and transcriptome analyses

**DOI:** 10.1186/s12920-022-01312-x

**Published:** 2022-07-13

**Authors:** Jie Ren, Qihang Yuan, Jifeng Liu, Lei Zhong, Hanshuo Li, Guangzhen Wu, Feng Chen, Qizhen Tang

**Affiliations:** 1 Department of Oncology, The First Affiliated Hospital of Dalian Medical University, Dalian, Liaoning China; 2 Department of General Surgery, The First Affiliated Hospital of Dalian Medical University, Dalian, Liaoning China; 3 Department of Urology, The First Affiliated Hospital of Dalian Medical University, Dalian, Liaoning China

**Keywords:** Transient receptor potential channels, Kidney renal clear cell carcinoma, Prognisis, Bioinformatics

## Abstract

Kidney renal clear cell carcinoma (KIRC) is among the major causes of cancer-caused mortality around the world. Transient receptor potential channels (TRPs), due to their role in various human diseases, might become potential drug targets in cancer. The mRNA expression, copy number variation, single-nucleotide variation, prognostic values, drug sensitivity, and pathway regulation of TRPs were studied across cancer types. The ArrayExpress and The Cancer Genome Atlas (TCGA) databases were used to retrieve KIRC samples. Simultaneously, training, internal, and external cohorts were grouped. In KIRC, a prognostic signature with superior survival prediction in contrast with other well-established signatures was created after a stepwise screening of optimized genes linked to TRPs using univariate Cox, weighted gene co-expression network analysis, multivariate Cox, and least absolute shrinkage and selection operator regression analyses. Subsequent to the determination of risk levels, the variations in the expression of immune checkpoint genes, tumor mutation burden, and immune subtypes and response between low-risk and high-risk subgroups were studied using a variety of bioinformatics algorithms, including ESTIMATE, XCELL, EPIC, CIBERSORT-ABS, CIBERSORT, MCPCOUNTER, TIMER, and QUANTISEQ. Gene set enrichment analysis helped in the identification of abnormal pathways across the low- and high-risk subgroups. Besides, high-risk KIRC patients might benefit from ABT888, AZD6244, AZD7762, Bosutinib, Camptothecin, CI1040, JNK inhibitor VIII, KU55933, Lenalidomide, Nilotinib, PLX4720, RO3306, Vinblastine, and ZM.447439; however, low-risk populations might benefit from Bicalutamide, FH535, and OSI906. Finally, calibration curves were used to validate the nomogram with a satisfactory predictive survival probability. In conclusion, this research provides useful insight that can aid and guide clinical practice and scientific research.

**Supplementary Information:**

The online version contains supplementary material available at 10.1186/s12920-022-01312-x.

## Introduction

Kidney cancer has long been recognized as one of the major death causes related to cancer around the world. Kidney renal clear cell carcinoma (KIRC) represents 80% of kidney malignancies [1]. Notably, KIRC lacks sensitivity to radiotherapy and chemotherapy. Despite the fact that targeted therapy is key in KIRC treatment, the prognosis remains dismal [2–4]. It is challenging to improve the treatment benefits owing to a lack of in-depth understanding of the KIRC-related underlying mechanisms. As a result, it is crucial to develop a novel prognostic signature that can help predict KIRC prognosis with more accuracy.

David Julius and Ardem Patapoutian received the Nobel Prize award in Physiology or Medicine in 2021 for discovering the touch and temperature receptors. Transient receptor potential channels (TRPs) were identified as the proteins that perceive these ubiquitous stimuli in their research. TRPs, a type of channel protein family, were found throughout the peripheral and central nervous systems [5, 6]. It was involved in sensory perception as well as cellular physiology. TRPs’ various physiological functions and regulatory mechanisms influenced the association between them and diseases, thus, targeting one or more of them had the potential to relieve the corresponding symptoms. Most of them served as the objects of drug discovery [7]. Early drugs for the TRPs focused on painkillers [8], and with a better understanding of the TRPs, new indications were expanded, including respiratory diseases, neurological and mental disorders [9, 10], diabetes, and cancers [11, 12]. The expression of some TRPs might change in cancer, but it was not clear whether this was the cause or result of the disease. However, cancers that were easy to administer and overexpress TRPs could be treated with potential therapeutic drugs targeting TRPs.

Nowadays, little is known about TRPs and their potential roles in cancers, and research has not been reported in KIRC. For this reason, the objective of this study is to investigate the possible involvement of TRPs across cancer types so that a novel TRP-related prognostic panel (TRPP) could be developed to differentiate high-risk and low-risk KIRC patients and demonstrate the possible discrepancies in individuals with various prognosis states. Lastly, a nomogram for predicting KIRC patient survival rates was established, which could be utilized to aid clinical decisions and personalized care. We are confident that the results of this study will offer new insight into the diagnosis and treatment of KIRC, in addition to providing a theoretical foundation for future TRP studies.

## Materials and methods

### Data collection

The Cancer Genome Atlas (TCGA) system was debuted by the National Human Genome Institute and the National Cancer and Cancer Institute in 2006 with the goal of mapping genes linked to cancer, in-depth study of cancer-related underlying mechanisms, and advancing the ability to prevent the progression of cancer, making accurate diagnoses, and treating malignancies. The ArrayExpress is an international open-source repository for academically published functional genomics data from microarray and sequencing technologies. The TCGA database was used to obtain single-nucleotide variation (SNV), transcriptome profiles, copy number variation (CNV), and clinical features of pan-cancer transcriptomes. The transcriptome profiles of KIRC patients, as well as their clinical characteristics, were also retrieved from the ArrayExpress database. Additionally, TRPs were summarized on the basis of the GENCODE (https://www.genecards.org/) platform and an article published in the “Nature Reviews Drug Discovery” journal in 2021 [7].

### Data procession

To find intersecting genes, we took the intersection of transcriptome profiles from TCGA and transcriptome profiles from ArrayExpress. Then, we transformed expression data of intersecting genes into the log2(x + 1) form. The 'sva' package in R was used for batch normalization.

### Pan-cancer analysis

Recently, limited research has been done for investigating the association between TRPs and malignancies. Hence, the differences in TRPs in various malignancies are not adequately described. SNV and CNV data (retrieved from the TCGA database) were evaluated and graphically presented as heatmaps to provide a pan-cancer summary of TRPs’ variations. In addition, a pan-cancer analysis of differential mRNA expression was carried out. Furthermore, we carried out an univariate Cox regression analysis between the mRNA expression and overall survival to determine the value of TRPs in prognoses of varying malignancies. R and TBtools were used to conduct all of these analyses [13].

In order to unveil the differential role of pathways influenced by TRPs in multiple types of human malignancies, single sample gene set enrichment analysis (ssGSEA) was utilized to compute TRPs scores in each sample of each tumor. Samples with the top and bottom 30% of TRPs scores were picked out respectively into two groups. Based on the transcriptome of the two groups, gene set enrichment analysis (GSEA) was applied for exploring the difference in pathway activities between the two groups. The IC50 of 265 small molecules in 860 cell lines as well as mRNA expression profiles of TRPs were obtained from Genomics of Drug Sensitivity in Cancer (GDSC). Pearson correlation analysis was then carried out to compute the correlation coefficients in pan-cancer between drug IC50 and mRNA expression profile of TRPs through the GSCA platform (http://bioinfo.life.hust.edu.cn/GSCA/#/).

### Creation, validation, and comparative analysis of the TRPP in KIRC

We focused on KIRC in this section for a thorough understanding. First and foremost, hub genes for panel construction were identified as follows: The R package, “limma”, was employed for screening differentially expressed genes (DEGs) between the KIRC samples and normal samples (filter criteria: |log2FC|> 2, FDR < 0.05); To screen for prognostic genes, we employed univariate Cox regression analysis adjusted by the Benjamini & Hochberg (BH) method (filter criteria: FDR < 0.05); DEGs with prognostic values were retained as candidate genes for weighted gene co-expression network analysis (WGCNA) to screen prognostic DEGs linked to TRP as real hub genes.

The WGCNA procedure was based on the R package “WGCNA”. The scale-free topology model was then determined using the soft threshold power β as 3 (scale-free R^2^ = 0.893). Subsequently, we converted the adjacency matrix to a topological overlap matrix (TOM). The dissimilarity metric as per TOM was utilized to separate the prognostic DEGs into various modules. Hub modules were defined as having a minimum module size of 30 and a cut height of 0.3. The module eigengene (ME) has been identified as the major principal gene module component. It can be taken as a module’s particular pattern of gene expression, the ME can summarize the gene expression profiles, and the association of ME with the expression level of TRPs was determined to screen the gene modules linked to TRPs.

Then, at random, 70% of samples of the KIRC in the TCGA database with total transcriptome data and survival time were chosen to establish a train cohort. Subsequently, the remaining 30% of KIRC samples were assigned to the test1 cohort as an internal validation. Meanwhile, all samples from the TCGA dataset were assigned to the test2 cohort as another internal validation, whereas all samples from the ArrayExpress dataset were assigned to the test3 cohort as external validation.

The least absolute shrinkage and selection operator (LASSO) regression analysis was employed in the train cohort for eliminating collinearity, preventing over-fitting, and choosing the most applicable variables from the above real hub genes. Subsequently, TRPP was created using multivariate Cox analysis between clinical outcomes and LASSO-derived hub genes’ mRNA expression, and each sample’s risk score was computed by the “predict” function in R. After the risk score calculation, the samples within the train cohort were classified into subgroups as per their median risk scores: low-risk and high-risk. As per the median risk scores acquired in the train cohort, all samples in the test1, test2, and test3 cohorts were sorted into low-risk and high-risk subgroups for subsequent study.

The train, test1, test2, and test3 cohorts were analyzed in the following steps for the internal and external validation of TRPP: (1) principal component analysis (PCA) was used for visualizing sample categorization; (2) the Fisher's exact test was utilized to investigate the variations in clinical features between the two subgroups in terms of the component; (3) a heatmap illustrating the TRPP-related genes’ expression levels was created using the R package “pheatmap”; (4) the Kaplan–Meier method was employed to conduct a survival study in order to see if the signature could be utilized to predict survival; (5) receiver-operating characteristic (ROC) curves on the basis of the area under the curve (AUC) were created to assess the risk score's diagnostic value; (6) In order to avoid the influence of different clinical characteristics, we also performed a survival analysis comparing high- and low-risk subgroups with the same clinical characteristics.

Using R software's “survival”, “survminer”, and “timeROC' packages, our TRPP was compared to seven other prognostic signatures (a mast cell-based signature developed by Liu et al. [14], an m5C-related risk signature developed by Wu et al. [15], and a four hypoxia-associated long non-coding RNA signature developed by Chen et al. [16], an m6A-lncRNAs prognostic index developed by Lin et al. [17], an autophagy-associated gene prognostic model developed by Wang et al. [18], a nine-RNA binding protein signature developed by Zhong et al. [19], and an inflammasome-related signature developed by Zheng et al. [20]) on the basis of each gene combination in each signature.

### Tumor mutation burden (TMB) analysis, GSEA, and drug sensitivity prediction

The tumor mutation burden (TMB) was defined as the total number of errors including somatic gene coding, deletion, gene insertion, and base substitutions identified per million bases. Non-synonymous mutations were counted utilizing the “perl” language. Then the difference in TMB across the low-risk and high-risk subgroups was computed with *p* value < 0.05 as the criteria for statistical significance and the association between TMB and the risk score was investigated utilizing the Spearman correlation coefficient. TMB analysis was limited to the train, test1, and test2 cohorts owing to TMB data lacking in the ArrayExpress dataset.

The Kyoto Encyclopedia of Genes and Genomes (KEGG) is well-known for providing functional annotations on various malignancies [21–23]. For KEGG analysis, the GSEA software (v4.1.0) was employed to find atypical pathways underlying the high-risk and low-risk subgroups [24, 25].

In addition to these, we employed the “pRRophetic” package in R to predict drug sensitivity for each KIRC patient, and the “Wilcox.test” function in R was utilized to screen the potentially sensitive drugs for high-risk and low-risk subgroups. Only drugs that were statistically significant in all the train, test1, test2, and test3 subgroups were considered true and reliable targeted drugs. Of note, the lower the IC50 value, the better the drug sensitivity.

### Immune checkpoint gene (ICG) expression, immune subtypes, and response differences between low-risk and high-risk subgroups

We studied the immune-related differences and their association with prognosis. First, we analyzed the differential expression of common ICGs in high-risk and low-risk subgroups, with only the statistically significant outcomes shown (*p* < 0.05). Each sample’s immune subtype in the train and test2 cohorts was then determined following a method published in the journal “Immunity” in 2018 [26]. Six immune subtypes were identified: (1) Wound healing (C1), (2) IFN-γ dominant (C2), (3) inflammatory (C3), (4) lymphocyte depleted (C4), (5) immunologically quiet (C5), and (6) TGF-b dominant (C6). The chi-square test was employed to explore the differences in immune subtype composition across different subgroups. We used the EPIC, XCELL, QUANTISEQ, MCPCOUNTER, CIBERSORT-ABS, CIBERSORT, and TIMER algorithms to compare immune responses in the high-risk and low-risk subgroups as per the TRPP for a thorough observation of immune components in the tumor microenvironment. Similarly, only statistically significant (*p* < 0.05) results were presented.

### Nomogram construction and validation

First, univariate and multivariate Cox regression analyses were employed to establish whether the risk score had independent prognostic significance. Subsequently, a nomogram was developed using the R package “rms” by integrating risk scores and other clinical parameters, and calibration curves for survival probability of 1–10 years were plotted to ascertain the degree of fit between the nomogram-predicted survival rates and the actual rates. The nomogram's diagnostic value was also compared to other clinical features on the basis of the AUC of ROC curves.

## Results

### Data procession

**Fig. 1:**
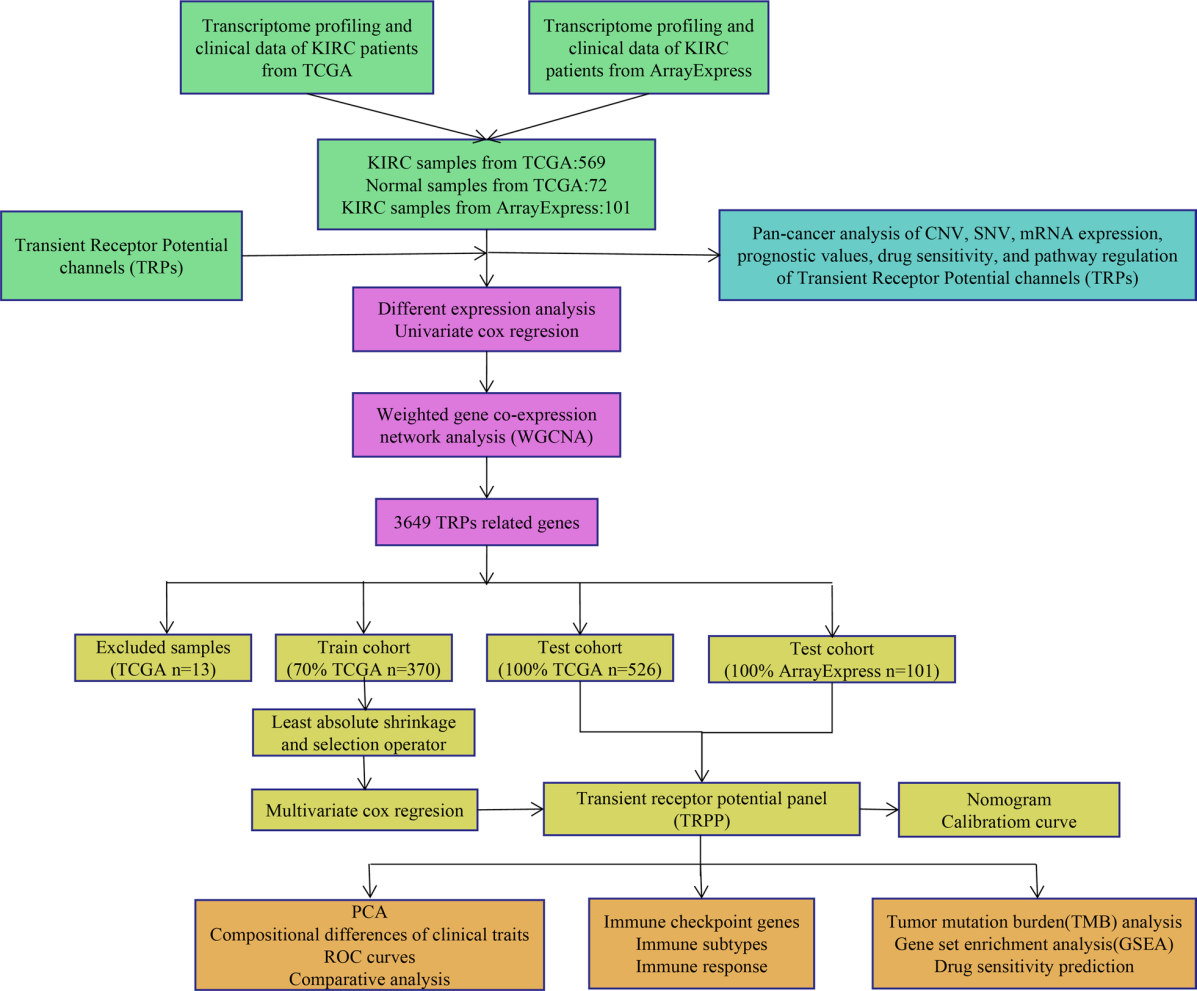
The current study’s workflow

A chart demonstrating the research steps is shown in Fig. 1. For the pan-cancer study, TCGA provided SNV, CNV, mRNA expression profiles, and survival data for 34 TRP signaling pathway genes in all kinds of malignancies. 539 KIRC samples and 72 normal samples with mRNA expression data of 56,753 shared genes were obtained from the TCGA database for specific analyses in KIRC, as well as 101 KIRC samples with mRNA expression data of 14,094 genes from the Array-express database (E-MTAB-1980). After excluding the samples with incomplete survival data, 526 TCGA KIRC samples and 101 ArrayExpress KIRC samples with the mRNA expression data of 13,005 shared genes were curated.

### Pan-cancer introduction regarding variations of TRPs

**Fig. 2:**
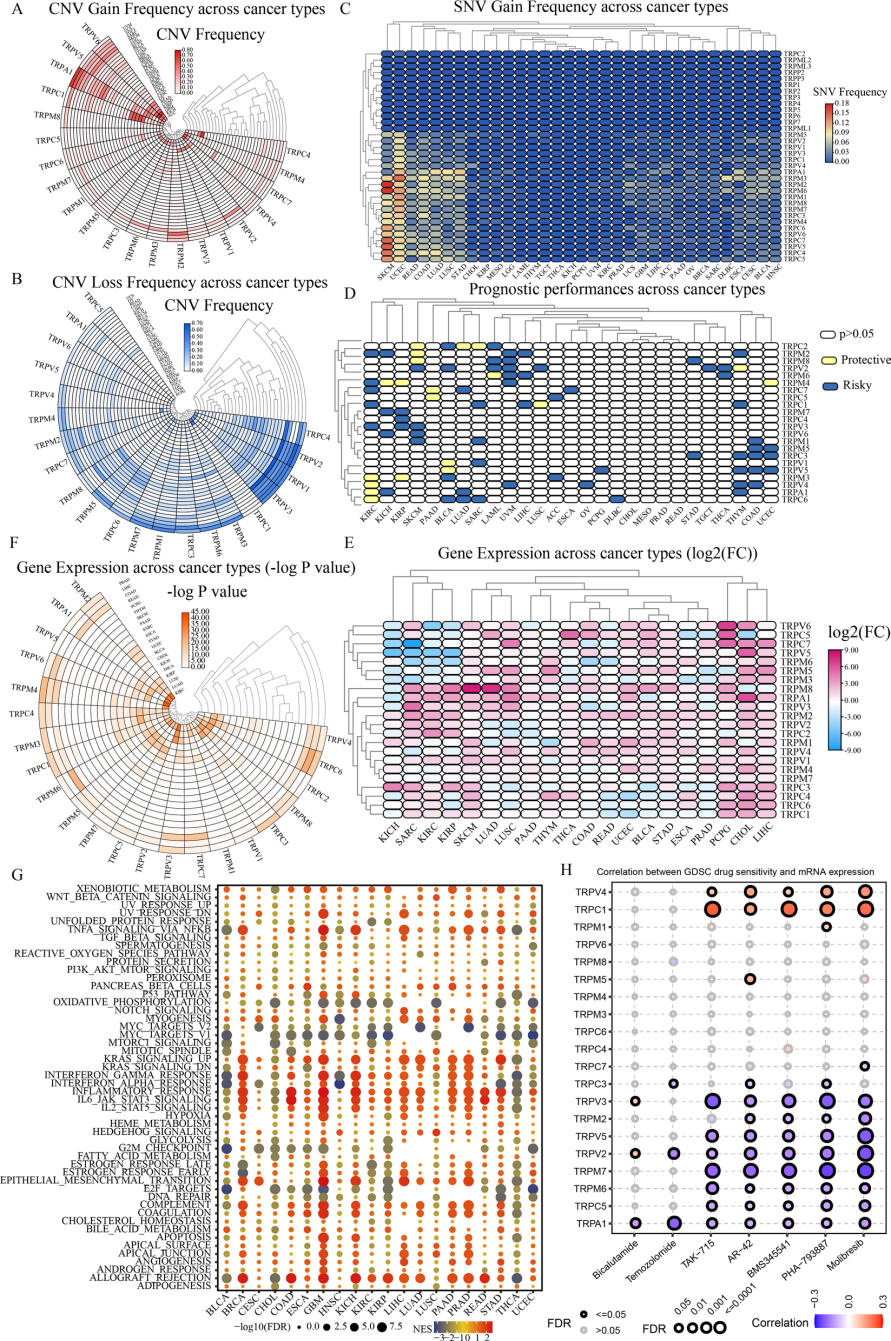
Panoramic view of TRP channels in pan-cancer. **A** The gain frequencies of Copy number variation (CNV) in various cancers. **B** CNV’s loss frequencies in a variety of cancers. **C** Pan-cancer-related Single-nucleotide variation (SNV). **D** TRPs’ survival profiles across cancers. **E** Variations in TRP mRNA expression across cancers (FC: Fold changes). **F** The relevant -logP value of each gene's variations across different cancers. **G** Enrichment analysis for cancer pathway signaling between tumor samples with high- and low-TRPs scores. (NES: normalized enrichment score; The redder the color, the higher the NES; The larger the dot, the lower the corrected *p*-value). **H** Relationship between TRPs expression and drug sensitivity based on the GSCA platform in pan-cancer (The darker the red color, the higher the Pearson’s correlation; The larger the dot, the lower the corrected *p*-value)

SNV and CNV data were shown as a heatmap for summarizing and visualizing the variation of TRP signaling pathway genes in diverse malignancies. The CNV gain frequency heatmap in Fig. 2A shows that TRPs have higher gain frequencies in UVM, USC, TGCT, OV, KIRP, KICH, and ACC. Gain variations were observed in the great majority of cancers for TRPV6, TRPV5, TRPA1, and TRPC1. The CNV loss frequency heatmap is depicted in Fig. 2B, showing that TRPs had greater frequencies of loss variations in THYM, KIRP, TGCT, LGG, UVM, and PCPG. TRPC4, TRPV2, TRPV1, and TRPV3 loss variations were found in nearly all cancers. Furthermore, the heatmap developed from the SNV data demonstrated that SKCM, UCEC, READ, COAD, LUAD, LUSC, and STAD all had remarkable SNV of TRPs (Fig. 2C).

### Pan-cancer analysis of the TRPs’ prognostic values and mRNA expression

As per current research in cancer, abnormal expression of mRNA may show that the gene of interest is likely to play a key role in disease progression [27–29]. Then, using univariate Cox regression of mRNA expression and overall survival (OS), risky TRPs with HR > 1 and *p* value < 0.05 as well as protective TRPs with HR < 1 and *p* value < 0.05 were identified, as shown in the Fig. 2D. Figure 2E indicates the mRNA expression levels for a visual exhibition. In the heat map, TRPC7 had simultaneous low expression in SARC; TRPM8 had remarkably increased expression levels in SKCM and LUAD; TRPV5 and TRPA1 had simultaneous increasing expression in CHOL; Up-regulation of TRPV6, TRPC5, and TRPC7 existed in PCPG. All of these conclusions were on the basis of |log2FC|> 2. A heatmap of the respective -lg (pValue) was constructed to more clearly demonstrate the significance of the difference in mRNA expression levels. The more orange the hue, the more pronounced the alteration in the expression of mRNA in cancer (Fig. 2F).

### TRPs-mediated pathway regulation and targeted drug prediction in human multiple cancers

At present, the regulatory effect of TRPs on cancer-related pathways is unknown, so it is very necessary to analyze the potential relationship between TRPs and these pathways, which will lay a foundation for the study of the regulatory mechanism of TRPs in pathways of pan-cancer. Our results showed that TRPs scores were positively correlated with xenobiotic metabolism, TNF-α signaling pathway, KRAS signaling pathway, inflammatory response, IL-2/STAT5 signaling pathway, epithelial-mesenchymal transition signaling pathway, IL-6/JAK/STAT3 signaling pathway, and complementary response in most types of tumors; however, they were negatively correlated with oxidative phosphorylation, MYC targets, mTOR signaling pathway, G2M checkpoints, E2F targets, and DNA repair (Fig. 2G). As illustrated in Fig. 2H, the GSCA platform determined 7 target drugs closely associated with TRPs, which might lay the foundation for accurate treatment and individual intervention of tumors.

### Creation and comparative analysis of the TRPP in KIRC

**Fig. 3:**
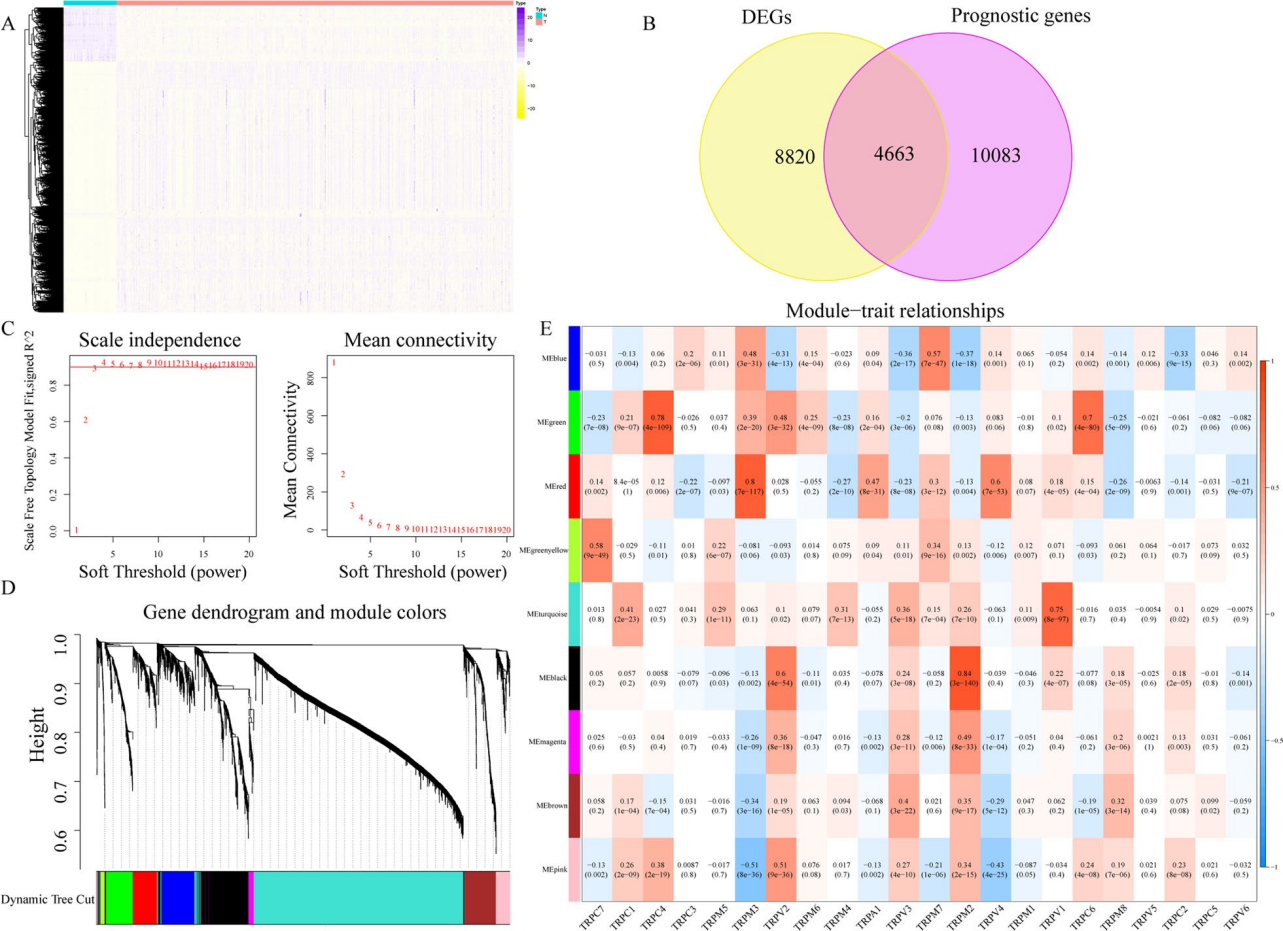
Identification of TRP-related prognostic DEGs in the TCGA dataset. **A** Heatmap to display mRNA levels of DEGs in KIRC and normal samples in the TCGA databset. **B** Venn diagram to find prognostic DEGs in the TCGA dataset. **C** Evaluation of the scale-free fit index for varying soft-thresholding powers (ß) and connectivity analysis of different soft-thresholding powers. **D** Dendrogram of DEGs clustered. **E** The association between module eigengenes and TRPs is depicted as a heatmap

In the subsequent KIRC-specific analysis, from the TCGA dataset, 539 KIRC and 72 normal samples were collated to conduct differential expression analysis and 13,483 DEGs were preserved (Fig. 3A, Additional file 1: Table S1). To carry out a univariate Cox regression analysis, 526 KIRC samples were obtained from the TCGA database with total mRNA expression and survival data, and 14,746 prognostic genes were selected (Additional file 1: Table S2). In total, 4663 DEGs with prognostic values were preserved as candidate genes (Fig. 3B).

Subsequently, the co-expression analysis was conducted to create a co-expression network. To meet a scale-free network with the scale-free R^2^ = 0.89, the power of β = 3 was selected (Fig. 3C). WGCNA revealed 9 co-expression modules using average linkage clustering (Fig. 3D). Of note, black, green, red, and turquoise modules showed a significant correlation with TRPs (cor > 0.7) (Fig. 3E). For further analyses, a total of 3649 genes in these 4 modules were considered real hub genes.

Following the exclusion of samples with incomplete clinical characteristics, 526 KIRC samples retrieved from the TCGA database and 101 KIRC samples retrieved from the ArrayExpress database (all having complete mRNA expression and survival data) were used to divide the samples into different cohorts. This study had four cohorts: the train cohort (370 TCGA samples, representing 70% of the total), test1 cohort (1560 TCGA samples, representing 30% of the total), test2 (all 526 TCGA samples), and test3 cohort (101 ArrayExpress samples). Specifically, during the TRPs validation, for the internal, the test1 and test2 cohorts have been used, whereas, for the external validation, the test3 cohort was used.

**Fig. 4:**
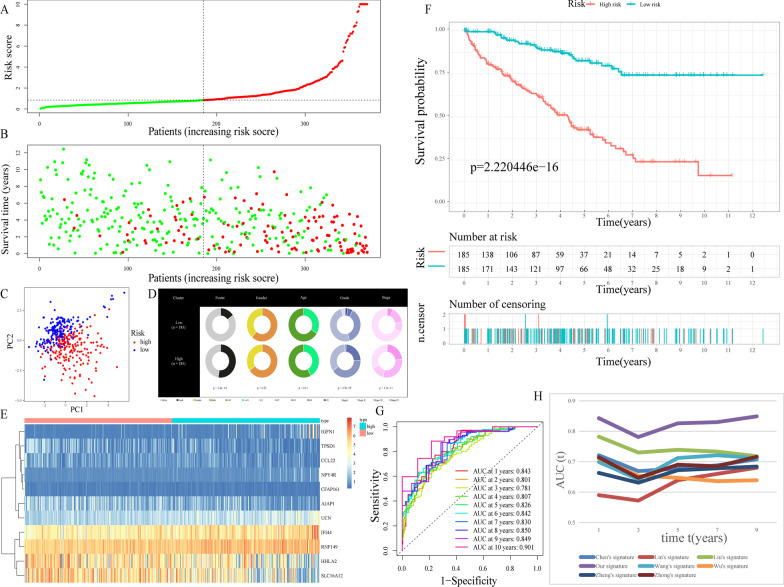
Construction of TRPP in the train cohort. **A** The train cohort was separated into different groups based on the median risk score. **B** The train cohort’s survival status and risk score distributions. **C** Train cohort’s PCA. **D** Compositional differences in clinical traits between the two subgroups. **E** A heatmap displaying the expression levels of 11 genes related to the TRPP in the train cohort. **F** Survival curve of the train cohort. **G** AUC values of ROC curves in the train cohort. **H** The AUC values of ROC curves for prediction ability of TRPP in comparison to seven additional well-established signatures in the train cohort

After performing LASSO regression and multivariate Cox analyses on the train cohort, 11 most applicable variables (i.e., AJAP1, IGFN1, CCL22, UCN, NPY4R, IFI44, HHLA2, TPSD1, CFAP161, RNF149, and SLC16A12) with prognostic values were identified from all genes in the four significant modules (Additional file 2: Fig. S1). On the basis of the expression data of these 11 genes and the results of multivariate Cox analysis, the “predict” function in R was applied to calculate risk score of each patient with KIRC in the train cohort (Additional file 2: Table S3). Following that, train cohort samples were separated into high-risk and low-risk subgroups based on the value of 0.843763 (the median risk score) (Fig. 4A). Patients having greater risk scores had a larger chance of dying, according to the risk score distributions and survival status (Fig. 4B). Patients from the high-risk and low-risk subgroups may be easily discriminated, according to the PCA data shown in Fig. 4C. As illustrated in Fig. 4D, the high-risk population possessed a higher proportion of G4 and stage IV samples and a lower proportion of G1, G2, and stage I samples, while the opposite was the case for the low-risk subgroup. In addition, in the high-risk subgroup, there are more dead patients (all *p* < 0.05). The expression levels of the 11 genes included in this model could be visualized using a heatmap as illustrated in Fig. 4E, which were likewise well correlated with the coefficients in the equation obtained from the multivariate Cox analysis. The genes IGFN1, UCN, NPY4R, IFI44, and RNF149 were found to be more significantly expressed in the high-risk subgroup in contrast with that in the low-risk subgroup. On the contrary, the genes AJAP1, CCL22, HHLA2, TPSD1, CFAP161, and SLC16A12 were expressed at lower levels in the high-risk subgroup. Individuals in the high-risk subgroup had a consistently decreased OS rate (*p* < 0.05), as shown in Fig. 4F. Moreover, the AUC values for the survival probability of the ROC curves of risk score vary from 0.781 to 0.901 for 1 to 10 years (Fig. 4G), implying that the risk score plays a substantial role in predicting survival for KIRC patients. Furthermore, when compared to seven other well-known prognostic markers, our TRPP exhibited a remarkably superior probability of predicting survival (Fig. 4H).

### Internal and external verification of the TRPP in KIRC

**Fig. 5:**
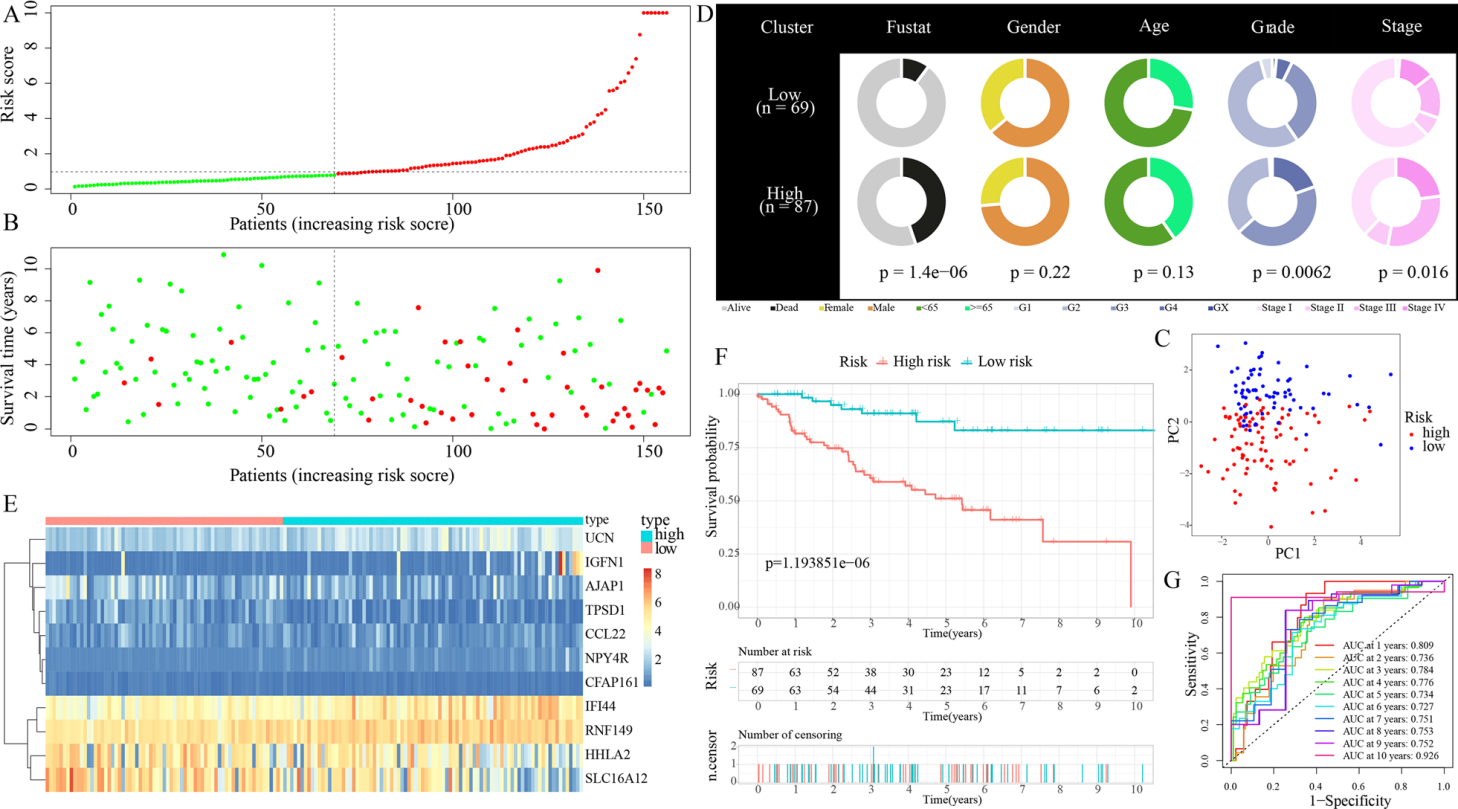
Internal verification of TRPP in test1 cohort. **A** There was a categorization of the test1 cohort into different subgroups. **B** The test1 cohort’s survival status and risk score distributions. **C** PCA of test1 cohort. **D** Compositional differences of clinical traits between the two subgroups in test1 cohort. **E** A heatmap displaying the expression levels of 11 genes linked to the TRPP in the test1 cohort. **F** Survival curve of the test1 cohort. **G** AUC values of ROC curves in the test1 cohort

**Fig. 6:**
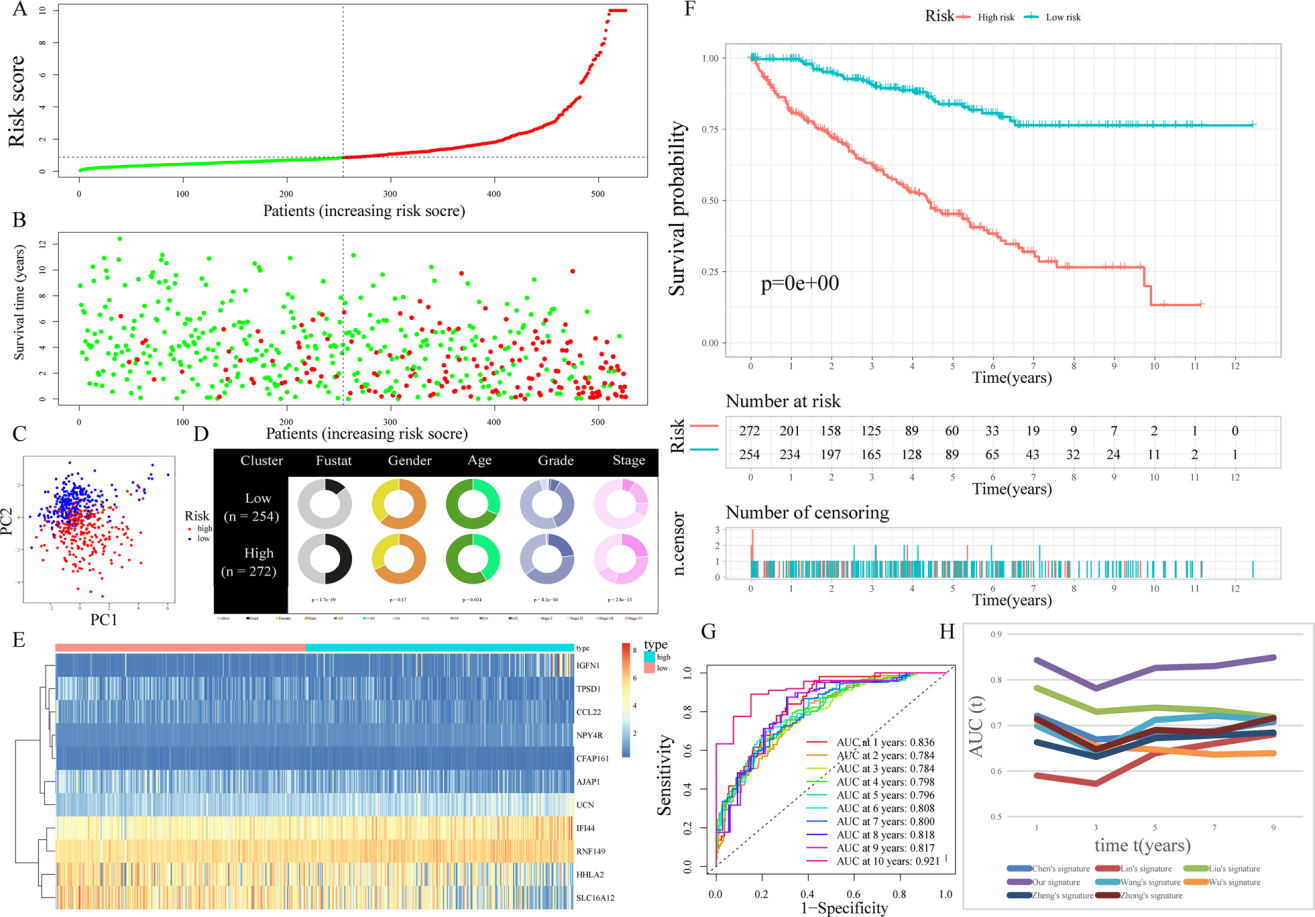
Internal verification of TRPP in test2 cohort. **A** The test2 cohort was classified into different subgroups. **B** The test2 cohort’s survival status and risk score distributions. **C** PCA of test2 cohort. **D** Compositional differences of clinical traits between the two subgroups in the test2 cohort. **E** In the test2 cohort, the heatmap depicts the expression levels of the 11 genes associated with the signature. **F** The test2 cohort’s survival curve. **G** AUC values of ROC curves in the test2 cohort. **H** The AUC values of ROC curves for the predictive performance of TRPP in comparison to seven additional signatures in the test2 cohort

**Fig. 7:**
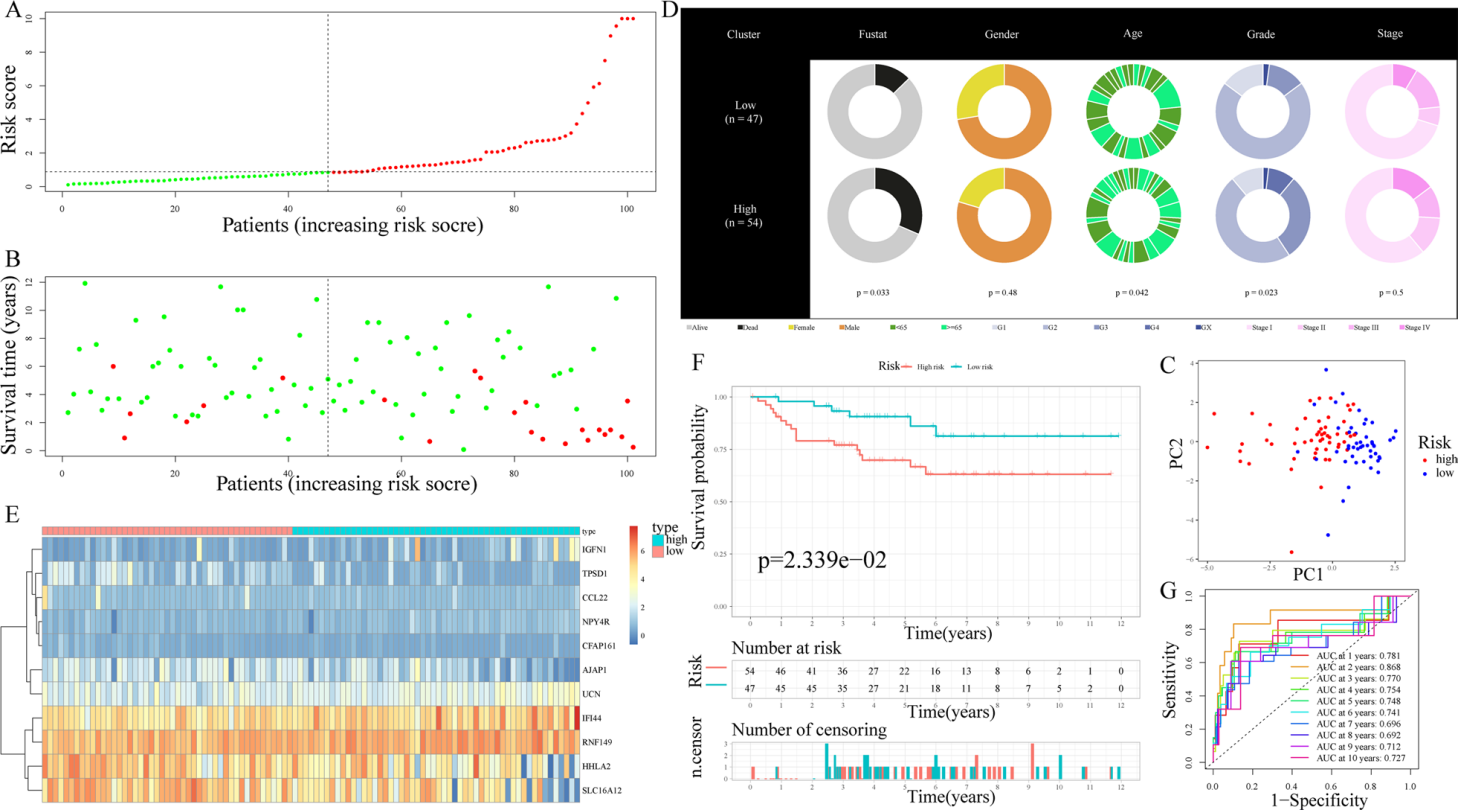
External verification of TRPP in test3 cohort. **A** The test3 cohort was classified into diverse subgroups. **B** The test3 cohort’s survival status and risk score distributions. **C** PCA of test3 cohort. **D** Compositional differences of clinical traits between the two subgroups in the test3 cohort. **E** Heatmap showing the expression levels of the 11 genes related to the TRPP in test3 cohort. **F** The test3 cohort's survival curve. **G** AUC values of ROC curves in the test3 cohort

Risk scores were produced and samples were separated into low-risk and high-risk subgroups for assessing the TRPP’s validity and reliability in test1, test2, and test3 cohorts respectively (Figs. 5A, 6A, 7A). Furthermore, the train cohort's median risk score (0.843763) served as a unified criterion for sorting the samples. The survival status and risk score distributions in both the internal (test1 and test2 cohorts) and external validation cohorts (test3 cohort) showed similar tendencies compared to the train cohort (Figs. 5B, 6B, 7B). PCA was used to show that patients could be easily distinguished from each other in the three subgroups (Figs. 5C, 6C, 7C). The discrepancies in clinical characteristics in low-risk and high-risk subgroups showed similar results (Figures 5D, 6D, 7D). In both internal and external validation cohorts, heatmaps created from the cohorts of test1, test2, and test3 suggested the existence of high-expression level genes (IGFN1, UCN, NPY4R, IFI44, and RNF149) and genes with attenuated expression (AJAP1, CCL22, HHLA2, TPSD1, CFAP161, and SLC16A12) in the high-risk subgroup (Figs. 5E, 6E, 7E). Furthermore, individuals having high-risk scores had poor OS in the internal as well as external validation cohorts (all *p* < 0.05) (Figs. 5F, 6F, 7F). The AUC of the risk score's ROC curves showed that the risk score's diagnostic value is excellent (Figs. 5G, 6G, 7G). Likewise, under the conditions of the same clinical characteristics, high-risk patients with KIRC had an increased likelihood of dying earlier compared to the low-risk individuals (Additional file 3: Fig. S2). Furthermore, when compared to seven other prognostic signatures in the test2 group, our TRPP exhibited obvious superiority in predicting survival (Fig. 6H).

### Tumor mutation burden (TMB) analysis, GSEA, and targeted drug prediction

**Fig. 8:**
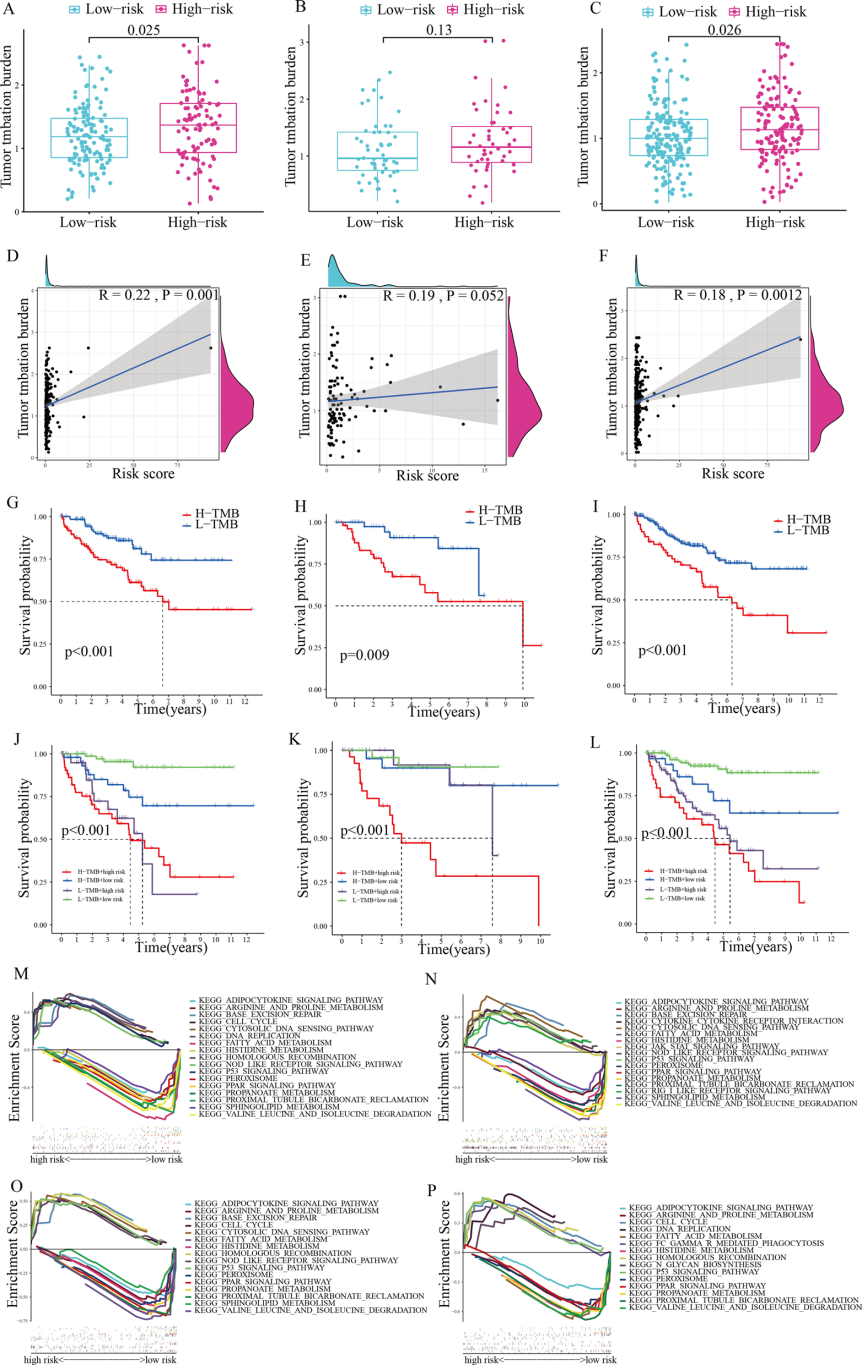
TMB analysis and GSEA (**A**–**C**) TMB difference between high- and low-risk subgroups in the train, test1, and test2 cohorts respectively. **D**–**F** Correlation of risk scores with TMB in train, test1, and test2 cohorts respectively. **G**–**I** The Kaplan–Meier curve of OS for patients in the train, test1, and test2 cohorts, shown by samples divided by TMB score. **J**–**L** The Kaplan–Meier curve of OS for patients in the train, test1, and test2 cohorts, shown by samples divided by both the risk score and TMB score. **M**–**P** GSEA of the low-risk and high-risk subgroups in train, test1, test2, and test3 cohorts

TMB was regarded as a novel biomarker for prognosis that is closely linked to immunotherapy response. Considering its crucial role in tumor therapy, we evaluated the correlation between TMB and risk score. Due to the lack of mutation data in the ArrayExpress database, the TMB analysis was only created for train, test1, and test2 cohorts, and not in the test3 cohort. In train, test1, and test2 cohorts, TMB was considerably elevated in the high-risk subgroup and its correlation with risk score was found to be positive (Fig. 8A–F). Additionally, patients who had low TMB levels had a longer survival time in comparison to those with high TMB levels (Fig. 8G–I). To further differentiate whether the TMB and risk scores are synergistic or antagonistic in predicting survival, we separated the patients on the basis of these two scores and did a survival analysis. There was a remarkable difference in survival between the two subgroups, with individuals with low TMB and risk scores having the best prognoses (Fig. 8J–L).

As for TRPP-based functional annotation, GSEA was utilized to observe the difference in pathway activity between the low-risk and high-risk subgroups, which could explain the prognosis difference. Base exclision repair, cell cycle, and homologous recombination, as shown in Fig. 8M–P, were enriched significantly in the high-risk subgroup, whereas proximal tubule bicarbonate reclamation, PPAR signaling pathway, adipocytokine signaling pathway, fatty acid metabolism, arginine and proline metabolism, histidine metabolism, peroxisome, sphingolipid metabolism, propanoate metabolism, and valine leucine and isoleucine degradation were found to be enriched in the low-risk subgroup in the train, test1, test2, and test3 cohorts.

**Fig. 9:**
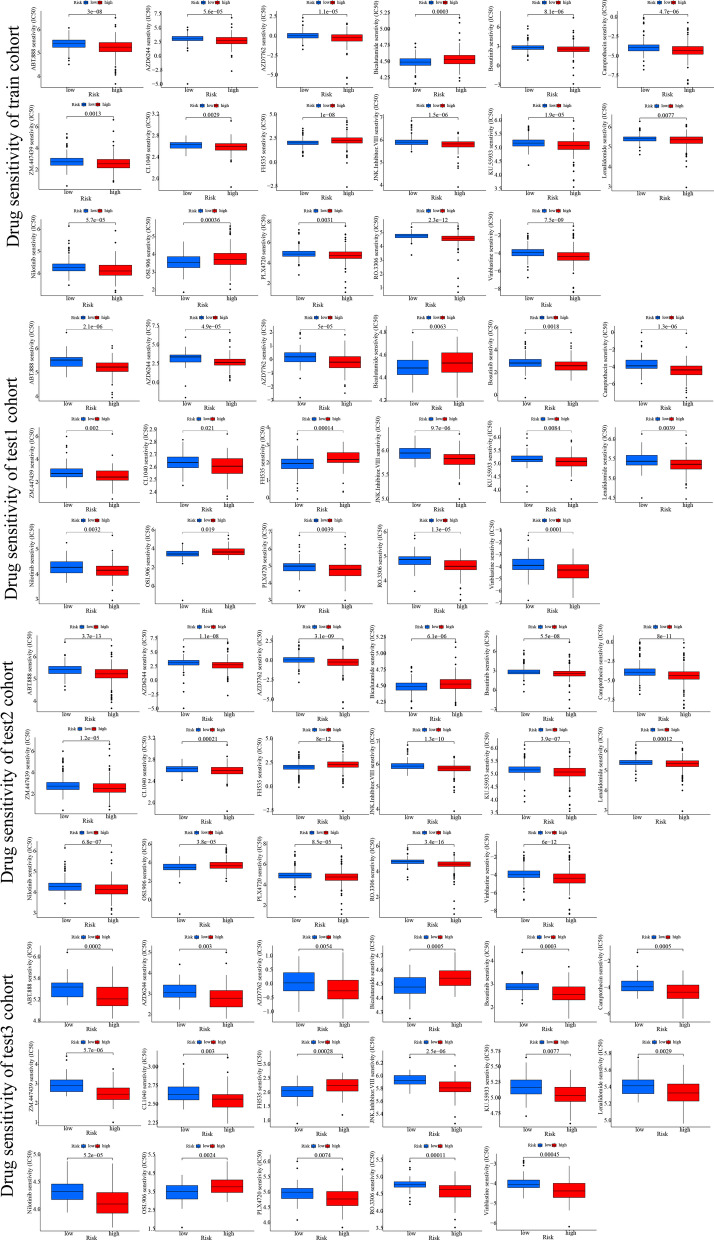
Relationship between TRPP and drug sensitivity. The box plots of the estimated IC50 for common chemotherapeutic agents (blue box plot represents low-risk populations; red box plot represents high-risk populations)

Considering the important role of molecularly targeted therapy in the prognosis improvement among KIRC patients, we used the R package “pRRophetic” to assess the expression profile characteristics of different risk populations to identify sensitive targeted therapeutic agents for low-risk and high-risk populations. Our findings reveal that KIRC patients that are at high risk might gain benefit from ABT888, AZD6244, AZD7762, Bosutinib, Camptothecin, CI1040, JNK inhibitor VIII, KU55933, Lenalidomide, Nilotinib, PLX4720, RO3306, Vinblastine, and ZM.447439; however, low-risk populations might benefit from Bicalutamide, FH535, and OSI906 in all the four cohorts (Fig. 9).

### ICG expression, immune subtypes, and response differences between high- and low-risk subgroups

**Fig. 10:**
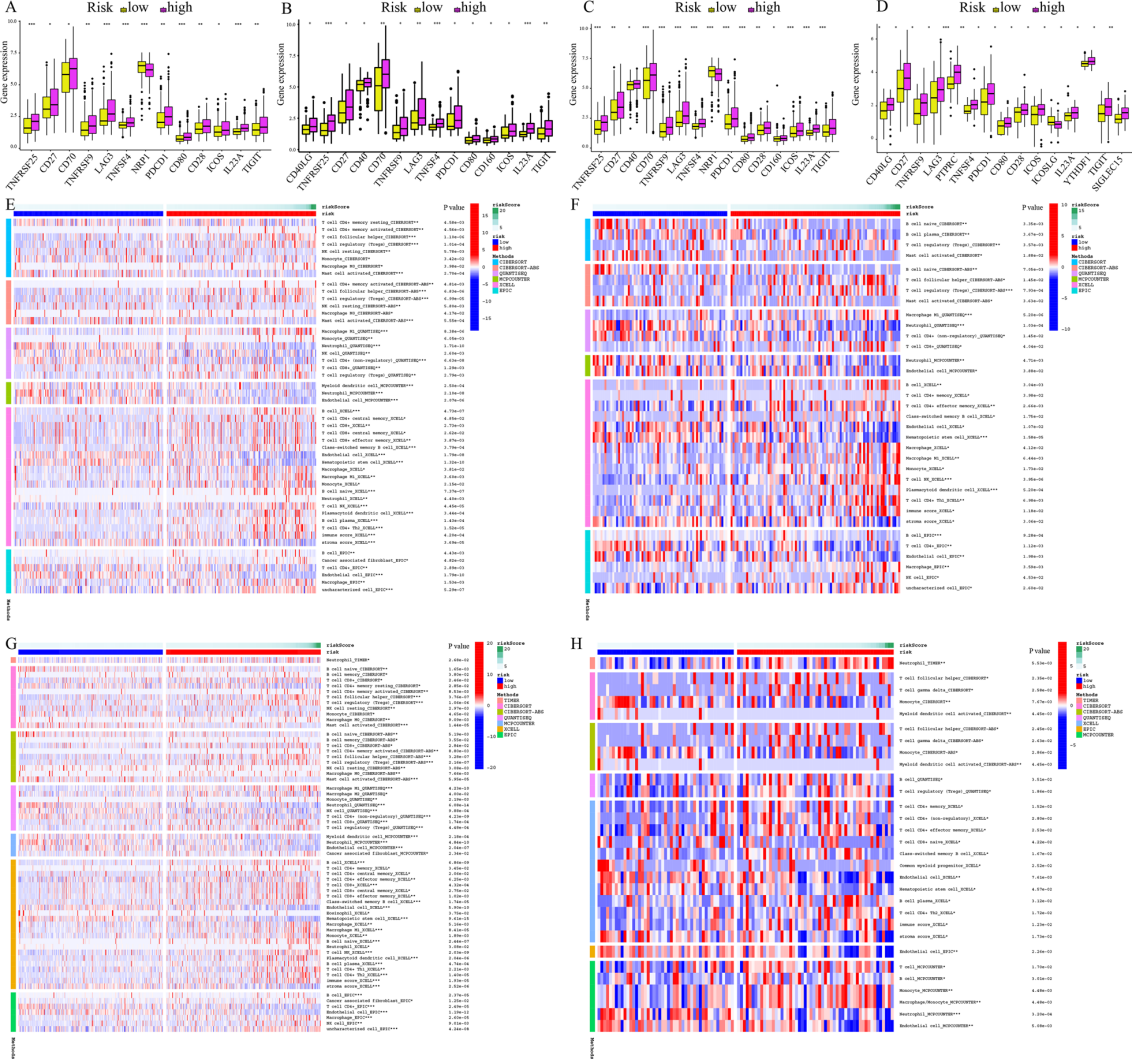
The discrepancies in the expression of immune checkpoint genes and abundance of immune cell infiltration in low-risk and high-risk populations. **A**–**D** Differential expression analysis of ICGs in the train, test1, test2, and test3 cohorts. **E**–**H** The landscape of immune cell infiltration in train, test1, test2, and test3 cohorts

The impact of varied ICG expression levels on the tumor immune microenvironment was studied. CD27, TNFRSF9, TNFSF4, PDCD1, CD80, ICOS, IL23A, and TIGIT were expressed at a higher level in the train, test1, test2, and test3 cohorts compared to the low-risk subgroup, whereas NRP1 in test2 and ICOSLG in test3 exhibited lowered expression levels in the high-risk subgroup (Fig. 10A–D). The difference in immune subtypes in train and test2 cohorts was identified for an in-depth study of the tumor immune microenvironment. The low-risk subgroup had a higher number of C3 subtype samples, whereas the high-risk subgroup had a higher number of samples of the C2, C4, and C6 subtypes (*p* < 0.001) (Additional file 4: Fig. 3A–B). Furthermore, In comparison to C3, C2 had more intratumor heterogeneity, SNV neoantigens, proliferation, and Th2 cells, and lesser Th17 cells, whereas C4 and C6 had lower lymphocytic infiltrates and higher M2 macrophage content [26]. The immunological responses were further investigated using the MCPCOUNTER, XCELL, QUANTISEQ, TIMER, CIBERSORT-ABS, and CIBERSORT algorithms, and visual heat maps were created. On the basis of XCELL, the immune score in the high-risk subgroup was higher in comparison to that in the low-risk subgroup. After finding the differences across the high- and low-risk subgroups in train, test1, test2, and test3 cohorts, it was found that numerous types of anti-tumor immune cells (e.g. activated CD4 + T cells, M1 macrophage, T follicular helper cells (Tfh), natural killer T cell (NKT), CD8 + T cells) had higher proportions but some cancer-promoting immune cells including T helper 2 (Th2), T cell regulatory (Treg), M2 macrophage, and plasmacytoid dendritic cell (pDC) are also upregulated in the high-risk subgroup (Fig. 10E–H).

### Nomogram development and verification

**Fig. 11:**
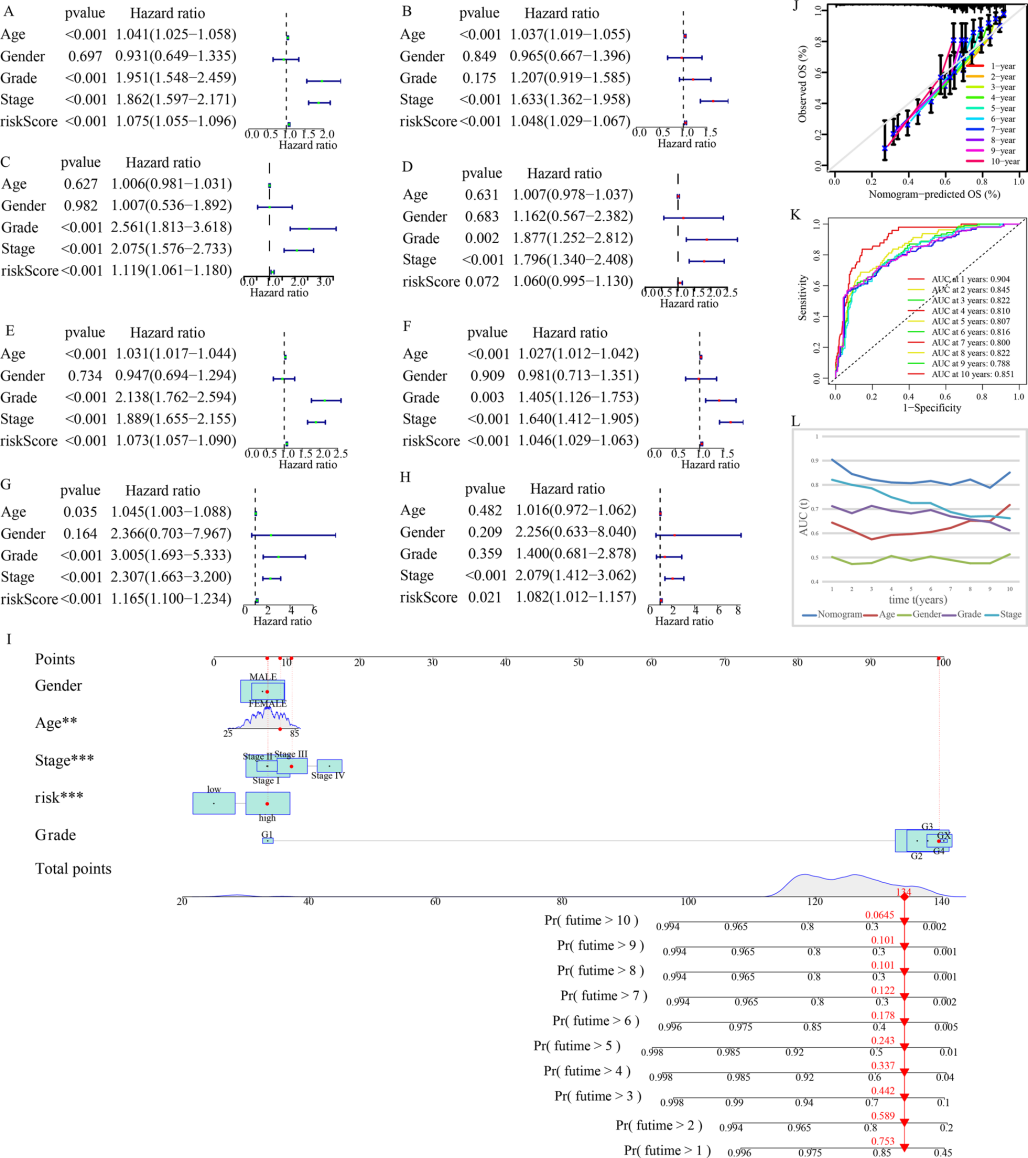
Risk score-based nomogram construction and verification. Univariate and multivariate Cox regression analyses in the train cohort (**A**, **B**), the test1 cohort (**C**, **D**), the test2 cohort (**E**, **F**), and the test3 cohort (**G**, **H**). (**I**) The nomogram for survival probability over 1–10 years. **J** Calibration curves used to verify the nomogram’s predictive ability. **K**–**L** Time-dependent ROC for overall survival predictions for the nomogram compared with different clinical indicators

According to the outcomes of the univariate Cox and multivariate Cox regression analyses, the risk score was found to be an independent prognostic predictor in the train cohort. Figure 11A–B shows all of the p-values and hazard ratios. Additionally, the risk score’s independent predictive role in KIRC was validated in the test1, test2, and test3 cohorts (Fig. 11C–H). After that, the above factors were integrated to create a nomogram. The total score may be simply determined based on the values of each variable for the OS prediction of KIRC patients over 1–10 years (Fig. 11I). Moreover, calibration curves were created for validating the nomogram’s anticipation power, and the result revealed the overall agreement between the nomogram-predicted survival rates and actual survival rates (Fig. 11J). The nomogram’s diagnostic value is better in comparison with other clinical parameters such as gender, age, grade, and stage, according to the AUC values of ROC curves of different clinical indicators (Fig. 11K–L).

## Discussion

As research into TRPs has progressed, the increasing roles of TRPs were discovered in cancer. Thus, we summarize variations of TRPs in a range of malignancies before studying the impact of aberrant TRPs in KIRC. In fact, TRPs’ variations more or less occurred and partial TRPs had prognostic values in various cancers. Moreover, the genetic mutations and alterations of TRPs were clearly observed in various cancers. A variety of signaling pathways including TNF-α, KRAS, IL-6/JAK/STAT3, IL-2/STAT5, epithelial-mesenchymal transition, oxidative phosphorylation, and mTOR were found to be significantly associated with TRPs in pan-cancer.

Then, to get an ideal signature with clinical significance, we analyzed prognostic DEGs linked to TRPs and tested the optimized candidate genes for signature construction. Subsequent to internal and external validation, a new TRPP with satisfactory prognostic performance was developed consisting of 11 genes (i.e., AJAP1, IGFN1, CCL22, UCN, NPY4R, IFI44, HHLA2, TPSD1, CFAP161, RNF149, and SLC16A12).

These eleven genes have been investigated in many types of cancers by other research studies. Some of them have been investigated in KIRC. The IGFN1 (Immunoglobulin-Like And Fibronectin Type III Domain Containing 1) gene is reported to have an impact on the formation of G-quadruplex structure so it could be targeted for therapeutic intervention in renal cell carcinoma [30]. C–C motif chemokine 22 (CCL22), a chemokine that acts on CCR4 + cells such as dendritic and T cells [31], participated in the progression of renal cell carcinoma and showed a connection with circ_0039569 and miR-34a-5p [32]. Urocortin (UCN), belonging to the corticotropin-releasing hormone (CRH) family, is involved in an immune-related signature in the clear cell renal cell carcinoma (ccRCC or KIRC) [33]. Interferon-induced protein 44 (IFI44), as one of the interferon-α-stimulated genes, was included in an Interferon Gamma response-related model and an apoptosis-related model for survival prediction in ccRCC [34, 35]. HHLA2, with the ability to hinder the human CD4 T-cells and CD8 T-cells proliferation and cytokine production [36], was regarded as a prognostic biomarker and therapeutic target in ccRCC [37–39]. Additionally, the adverse impact on the prognosis of HHLA2 and PD-L1 co-expression was discovered in ccRCC [40]. In ccRCC, the mRNA expression level of SLC16A12 was elevated in patients with higher TNM stages and poorer differentiated grades, whereas SLC16A12 immunoreactivity was predominantly found in the cytoplasm [41]. As for AJAP1, NPY4R, TPSD1, CFAP161, and RNF149, this research proposes the potential role of these five genes in KIRC for the first time. Adherens junctions-associated protein 1 (AJAP1 or Shrew-1), was originally found in epithelial cells and confirmed to be implicated in glioma [42], hepatocellular carcinoma [43], and esophagus carcinoma [44]. There are presently no data on the role of NPY4R in cancer, despite the fact that genetic and structural variation within the NPY4R gene has been linked to obesity onset [45]. A particular mutation of TPSD1 p.Ala92Thr was observed in colon cancer among those non-responders after 5-fluorouracil-based therapy [46]. CFAP161, located on chromosome 15q in the linkage region of Kartagener syndrome [47], was only studied in mice and Xenopus [48]. RNF149, demonstrated as a DEG between normal tissue and prostate cancer [49], was discovered to be associated with 2[18F]fluoro-2-deoxy-d-glucose (FDG) uptake during positron emission tomography (PET) and survival in non-small cell lung cancer patients [50].

With the help of TPRP, KIRC patients may be effectively categorized into the high-risk subgroup with a poorer prognosis and the low-risk subgroup with a better prognosis in the train, test1, test2, and test3 cohorts. Given the potential influence of the tumor immune microenvironment on tumor therapy, the following section examined the discrepancy in immune status between high-risk and low-risk subgroups of KIRC. Cancer cells are known to be mistaken as normal components of the human body, allowing for self-protection via immune checkpoint pathways. The high-risk subgroup having a greater percentage of immunological components had a worse prognosis, suggesting that immune checkpoint pathways were engaged. In both subgroups, immune checkpoint genes are subjected to differential expression. The upregulation of CD27, TNFRSF9, TNFSF4, PDCD1, CD80, ICOS, IL23A, and TIGIT and downregulation of NRP1 and ICOSLG might become promising targets in KIRC. The discrepancies in immune subtypes support the different prognoses in the two subgroups. Characterized with a comparatively lowered level of cancer-promoting Th2 cells, a higher number of C3 subtype samples belonged to the low-risk subgroup. However, the high-risk subgroup had a higher number of samples of the C2, C4, and C6 subtypes. C2 had more intratumor heterogeneity, SNV neoantigens, proliferation, Th2 cells, and lesser Th17 cells, whereas lower lymphocytic infiltrate with higher M2 macrophage content was displayed in C4 and C6. Furthermore, the abundance of immunocyte infiltration varied across high-risk and low-risk subgroups. The up-regulation of cancer-promoting immune cells including T helper 2 (Th2) [51, 52], T cell regulatory (Treg) [53], M2 macrophage [54], and plasmacytoid dendritic cell (pDC) [55] in the high-risk subgroup might be responsible for poor prognoses. More importantly, our TRPP also provides great aid for the accurate treatment of patients with KIRC. Specifically, many drugs, including Bosutinib, Camptothecin, JNK inhibitor VIII, Lenalidomide, Nilotinib, and Vinblastine, are more suitable for high-risk populations, while Bicalutamide, FH535, and OSI906 target drugs are more suitable for low-risk populations.

Additionally, TRPP, in comparison to other well-known signatures, has a better prediction performance for KIRC patients, and the risk score has been proven as an independent prognostic predictor. In order to fully exploit TRPP's prognostic potential, the survival rate of KIRC patients was quantitatively assessed after constructing a nomogram on the basis of risk score and other clinical features. The nomogram's predictive potential to anticipate with high accuracy was tested using calibration curves and ROC curves.

The current report had certain limitations that must be considered. First, as a result of discrepancies between the transcriptome profiles from the ArrayExpress database and that obtained from the TCGA database, TMB analysis, and immune-related analyses were only conducted with the TCGA data from the train, test1, and test2 cohorts. Concerning the validation of this nomogram, obtaining external validation might well be preferable. Moreover, the TRPP was constructed incorporating a small number of KIRC patients from the ArrayExpress and TCGA datasets. To establish the predictive significance of this prognostic signature, a larger prospective clinical research is required. Lastly, the TRPP was created only through bioinformatics research, implying that further fundamental investigations are necessary to support our findings.

## Conclusions

In this study, for the first time, a TRPP was successfully built and validated to reliably predict the KIRC patients’ prognoses. TMB, pathway activity, ICGs, and immune response differences were studied in patients with varying prognoses. Following that, using this panel and other clinical features, a nomogram was constructed as a quantitative tool to help the survival rate predictions for KIRC patients. To summarize, the current study may provide fresh insight into clinical decision-making and tailored therapy for KIRC patients.

## Supplementary Information

**Additional file 1.** **Table S1**: The results of differentially expressed genes between 539 KIRC samples and 72 normal samples from the TCGA database. **Table S2**: The results of univariate Cox regression analysis of 526 KIRC samples from the TCGA database. **Table S3**: The results of multivariate Cox regression analysis.

**Additional file 2.** **Figure S1**: Variable selection. **A**–**C** Eleven genes were selected by LASSO-Cox regression analysis.

**Additional file 3.** **Figure S2**: **A**–**C** Survival analysis between high- and low-risk groups with the same clinical characteristics in train, test1, and test2 cohorts.

**Additional file 4.** **Figure S3**: The discrepancies in immune subtypes in high-risk and low-risk populations. **A**–**B** The distribution of immunological subtypes (which include C1, C2, C3, C4, C5, and C6) across subgroups based on risk-score in the train and test2 cohorts is depicted in a heat map and a table.

## Data Availability

The datasets generated and analysed during the current study are available in the ArrayExpress and TCGA databases [https://portal.gdc.cancer.gov/; https://www.ebi.ac.uk/arrayexpress/].
