# Supplementary figures and images for: Identifying the role of transient receptor potential channels (TRPs) in kidney renal clear cell carcinoma and their potential therapeutic significances using genomic and transcriptome analyses
Source: BMC Med Genomics. 2022 Jul 13;15:156. doi: 10.1186/s12920-022-01312-x (PMC9277847; doi:10.1186/s12920-022-01312-x)

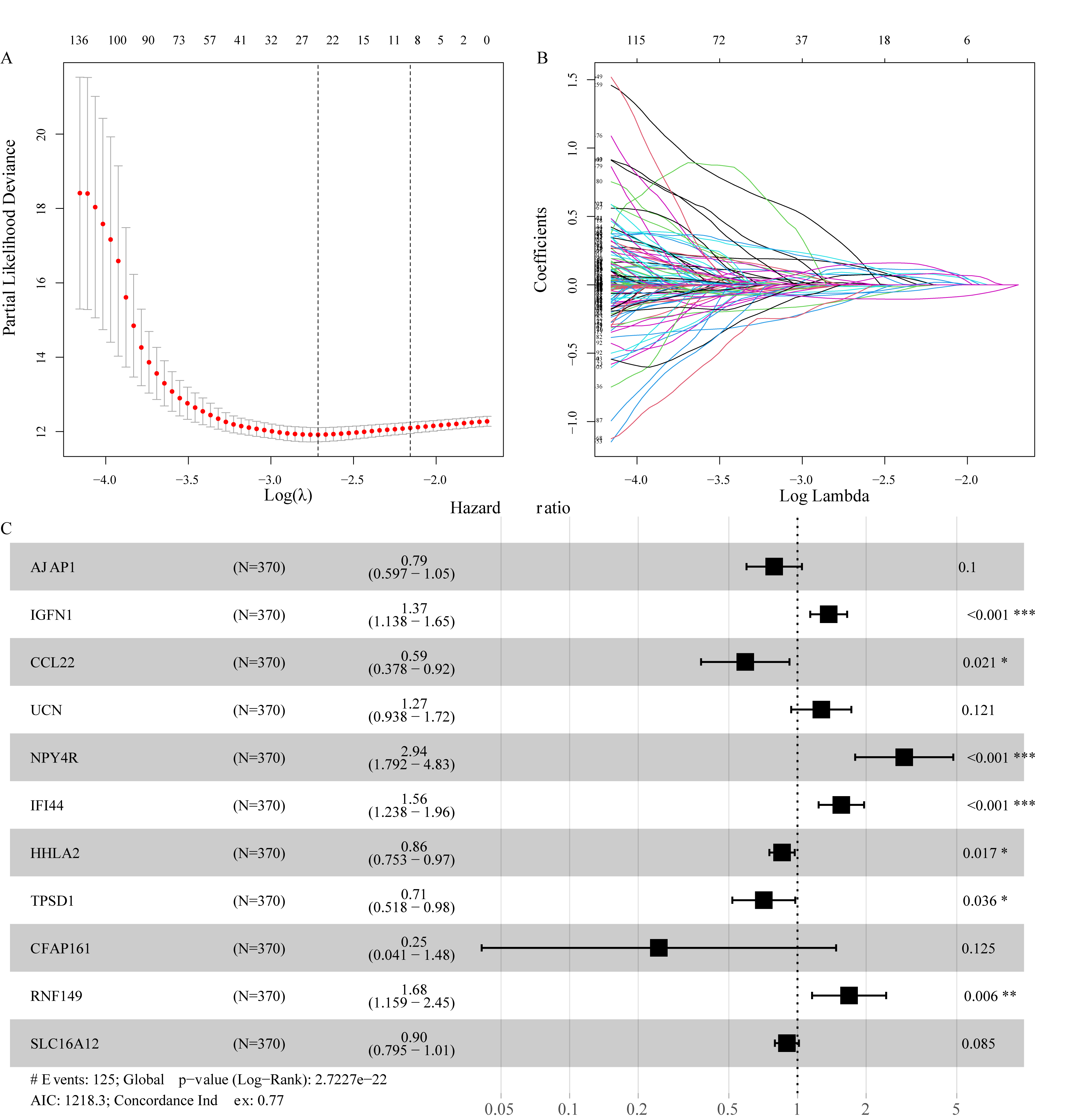

Supplement: Supplementary file 2 — Additional file 2. Figure S1: Variable selection. A–C Eleven genes were selected by LASSO-Cox regression analysis. [file 12920_2022_1312_MOESM2_ESM.tif]

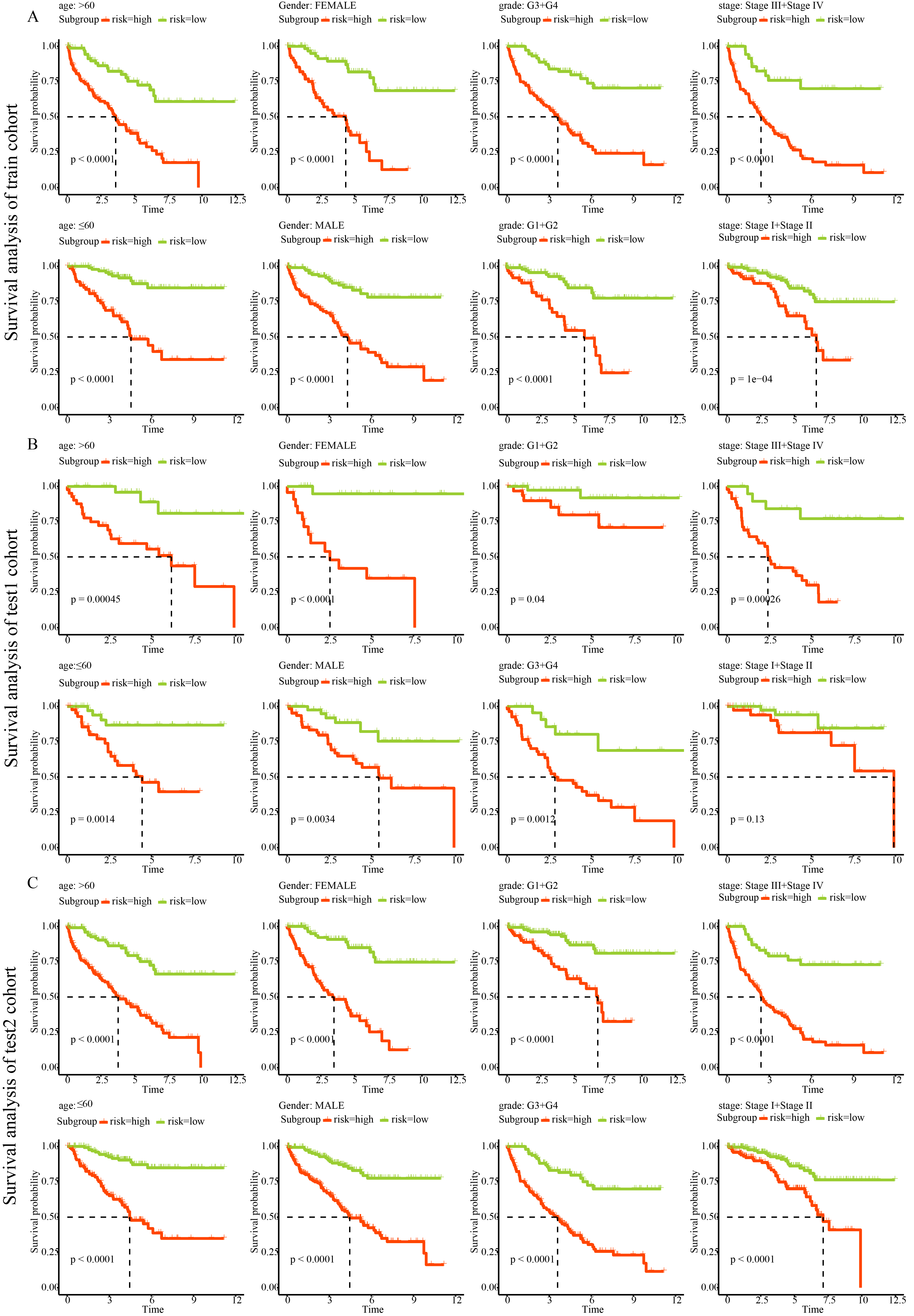

Supplement: Supplementary file 3 — Additional file 3. Figure S2: A–C Survival analysis between high- and low-risk groups with the same clinical characteristics in train, test1, and test2 cohorts. [file 12920_2022_1312_MOESM3_ESM.tif]

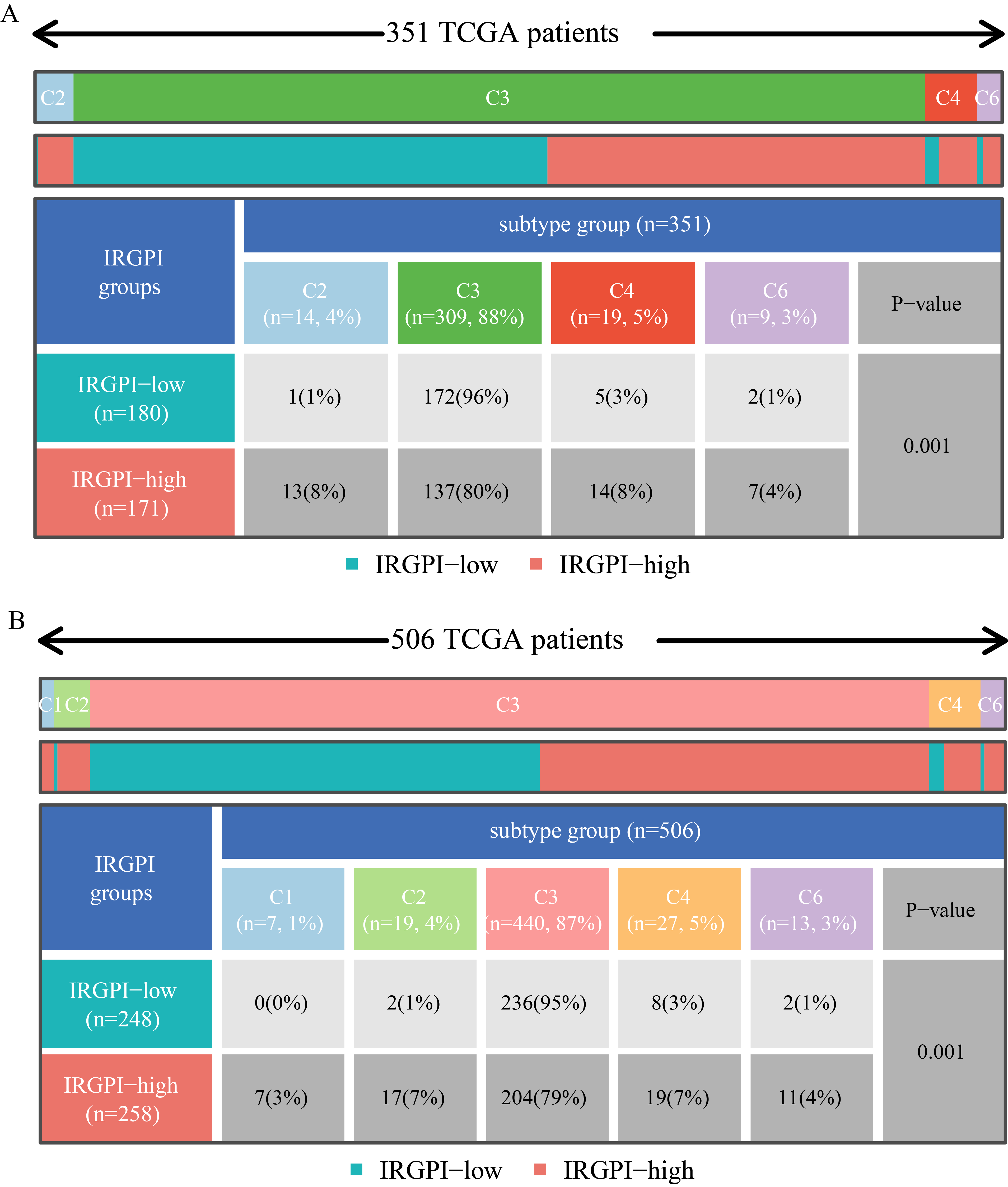

Supplement: Supplementary file 4 — Additional file 4. Figure S3: The discrepancies in immune subtypes in high-risk and low-risk populations. A–B The distribution of immunological subtypes (which include C1, C2, C3, C4, C5, and C6) across subgroups based on risk-score in the train and test2 cohorts is depicted in a heat map and a table. [file 12920_2022_1312_MOESM4_ESM.tif]
